# Peri-prostatic Fat Volume Measurement as a Predictive Tool for Castration Resistance in Advanced Prostate Cancer

**DOI:** 10.1016/j.euf.2017.01.019

**Published:** 2018-12

**Authors:** Mark Salji, Jane Hendry, Amit Patel, Imran Ahmad, Colin Nixon, Hing Y. Leung

**Affiliations:** a Department of Urology, NHS Greater Glasgow and Clyde, Glasgow, Scotland, UK; b CRUK Beatson Institute, Glasgow, Scotland, UK; c Institute of Cancer Sciences, University of Glasgow, Glasgow, Scotland, UK; d Department of Radiology, NHS Greater Glasgow and Clyde, Glasgow, Scotland, UK

**Keywords:** Peri-prostatic fat, MRI, Resistance, ADT, Visceral fat

## Abstract

**Background:**

Obesity and aggressive prostate cancer (PC) may be linked, but how local peri-prostatic fat relates to tumour response following androgen deprivation therapy (ADT) is unknown.

**Objective:**

To test if peri-prostatic fat volume (PPFV) predicts tumour response to ADT.

**Design, setting, and participants:**

We performed a retrospective study on consecutive patients receiving primary ADT. From staging pelvic magnetic resonance imaging scans, the PPFV was quantified with OsirixX 6.5 imaging software. Statistical (univariate and multivariate) analysis were performed using R Version 3.2.1.

**Results and limitations:**

Of 224 consecutive patients, 61 with advanced (≥T3 or N1 or M1) disease had (3-mm high resolution axial sections) pelvic magnetic resonance imaging scan before ADT. Median age = 75 yr; median PPFV = 24.8 cm^3^ (range, 7.4–139.4 cm^3^). PPFV was significantly higher in patients who developed castration resistant prostate cancer (CRPC; n = 31), with a median of 37.9 cm^3^ compared with 16.1 cm^3^ (p < 0.0001, Wilcoxon rank sum test) in patients who showed sustained response to ADT (n = 30). Multivariate analysis using Cox proportional hazards models were performed controlling for known predictors of CRPC. PPFV was shown to be independent of all included factors, and the most significant predictor of time to CRPC. Using our multivariate model consisting of all known factors prior to ADT, PPFV significantly improved the area under the curve of the multivariate models receiver operating characteristic analysis. The main study limitation is a relatively small cohort to account for multiple variables, necessitating a future large-scale prospective analysis of PPFV in advanced PC.

**Conclusions:**

PPFV quantification in patients with advanced PC predicts tumour response to ADT.

**Patient summary:**

The amount of fat around the prostate predicts prostate cancer response to hormone treatment.

## Take Home Message

Peri-prostatic fat volume is greater in patients who go on to develop relapsed cancer following hormonal therapy. Such measurements may complement the existing tools for predicting treatment response in patients undergoing androgen deprivation therapy.

## 1 Introduction

Prostate cancer (PC) is the most common cancer of men in the developed world. Treatment for symptomatic advanced or metastatic PC includes androgen deprivation therapy (ADT). All men receiving ADT eventually develop castration resistant prostate cancer (CRPC) [1]. Despite recent advances in second line chemotherapeutics, the median survival has only improved by 2–5 mo [2], [3], [4]. Upfront docetaxel therapy has shown a greater increase in survival when given with ADT, showing an increase in survival of 13.6 mo over ADT alone [5], [6]. An initial poor response to ADT with nadir prostate-specific antigen (nPSA) not falling below 4 mg/ml is a predictor of early CRPC [1]. As both nPSA and time to nPSA can only be calculated after ADT, there is an urgent need for clinically impactful predictive markers for the development of CRPC.

Epidemiological studies suggest an association between higher body fat (as estimated by body mass index; BMI) and earlier age of diagnosis and the risk of disease progression, including CRPC [7], [8], [9]. Visceral fat is metabolically active and may contribute to tumour promoting signalling in PC. Intriguingly, adipocyte-mediated chemokine secretion as part of a chemokine axis involving chemokine ligand 7 (CCL7) and its receptor chemokine receptor 3 (CCR3) may mediate interactions between local peri-prostatic fat (PPF) and PC to promote tumour progression [10], [11], [12]. Until recently, evaluation of PPF as a predictive marker has been limited to the analysis of either PPF thickness or area (ie, 1- or 2-dimension measurements) in patients undergoing radical prostatectomy or radical radiotherapy [13], [14], [15], [16]. By accurately quantifying the volume of PPF in patients with advanced PC prior to primary ADT, we tested if measuring PPF volume improves the current prognostic tools in identifying patients who are at risk of developing CRPC, and stratifies patients for enhanced monitoring or additional therapy from the outset.

## 2 Patients and methods

### 2.1 Patient selection

Over a 2-yr period, consecutive patients with locally advanced or metastatic PC treated with primary ADT were identified (n = 224). Patients underwent staging investigations by computed tomography (n = 151) or magnetic resonance imaging (MRI; n = 73) of the prostate and pelvis for local disease staging along with isotopic bone scan. Of 73 patients with MRI, the following patients were excluded for analysis: two due to lack of follow up (moving out of region), one patient with T2 localised disease, and nine with MRI after starting ADT. The majority were treated with luteinising hormone-releasing hormone analogues (n = 53) but also androgen receptor blocker alone (n = 4), oestrogen patches (n = 3), or luteinising hormone-releasing hormone antagonist (n = 1) were included.

### 2.2 Staging MRI analysis

Cases with T2-weighted pelvic MRI images at slice thicknesses of 3 mm or less were studied. Peri-prostatic fat volume (PPFV) was calculated by consecutive areas through the prostate, with the prostatic and seminal vesicular volumes subtracted to give the PPFV (see details below). Training for the recognition of PPF on T2-weighted MRI images was provided by a single uro-radiologist experienced in prostate MRI. Regions of interest were drawn on consecutive axial images by a single investigator using OsiriX (Version 6.5, 2013 Pixmeo Sarl, Switzerland). Waist circumference was measured using whole pelvis T2 axial images at vertebral level L5.

### 2.3 PPFV measurement technique

PPFV measurements were blinded to CRPC status and made by initially delineating the prostate gland and seminal vesicle. The surrounding region of PPF including vessels draining the prostate were then delineated laterally by the first visible fascial boundary adjacent to the levator muscles, posteriorly by Denonvillier’s facia (excluding the mesorectal fat) and anteriorly to the pubic symphysis, including the anterior venous plexus and retropubic fat, on each 3-mm slice from the bladder neck superiorly to prostate apex inferiorly. This delineated the region of surrounding adipose tissue of similar signal and composition including local vasculature providing venous drainage of the prostate and seminal vesicles. Collectively, these represent the local visceral adipose-rich tumour macro-environment.

### 2.4 Immunohistochemistry

Patients were grouped by their response to ADT into three groups: (1) Favourable Sustained Response to ADT (SRADT) with PSA remaining at nadir levels and below 4 ng/ml throughout the follow up period, (2) Initial Response to ADT (IRADT) with PSA falling below 4 ng/ml but then developing CRPC, and (3) Poor Response to ADT (PRADT) with nPSA not falling below 4 ng/ml and subsequently developing CRPC. Formalin fixed paraffin embedded prostate tumour sections were obtained in accordance with appropriate ethics approval (National Health Service Research Ethics Committee permission: 12/EE/0058), including SRADT (n = 6), IRADT (n = 6), and PRADT (n = 6). Sections were stained for CCR3 as a secondary outcome measure of PPFV influence on prostatic epithelium (rabbit monoclonal antibody; Abcam, ab32512 1:50 dilution), and signal amplified with Dako Rabbit EnVision (K4003) and visualised using Dako Liquid 3,3′-diaminobenzidine (DAB; K3468). Using the Leica Biosystems Image Analysis suit (v4.0.6), DAB staining was quantified by normalising the area of DAB staining to the area of haematoxylin counterstain.

### 2.5 Data collection and statistical analysis

Demographic data including age, initial PSA, nPSA, evidence of (distant and/or regional nodal) metastasis at the time of diagnosis, and time-to-reach nPSA were recorded. Biochemical progression to CRPC as the primary outcome measure was indicated by three successive rises in PSA level after commencing ADT [17]. Where available Gleason score, tumour stage, and presence of distant metastases and lymph node status were included. Statistical analysis were performed in R (Version 3.2.1) with significance taken as p < 0.05 and indicated by *, p < 0.001 **, p < 0.0001***. Univariate Cox proportional hazards models of time to CRPC were performed for PPFV, metastasis (lymph node and/or distant) at diagnosis, T stage, Gleason score, initial PSA, nPSA, body weight, and waist circumference. Multivariate Cox proportional hazards models of time to CRPC were performed with covariates PPFV, metastasis (lymph node and/or distant) at diagnosis, T stage, Gleason score, initial PSA, and body weight, excluding nadir PSA as this can only be known after commencing ADT.

## 3 Results

Between January 2011 to December 2012, 224 patients receiving primary ADT were identified. Among them, 61 patients had an MRI of the prostate and pelvis prior to commencing ADT (median of 23 d prior to ADT). The remaining 163 patients were excluded due to the use of a staging computed tomography instead of MRI (n = 151), T2 localised disease (n = 1), lack of follow-up data due to moving out of region (n = 2), or the MRI being performed after commencing ADT (n = 9). Of the 61 patients analysed (median age 75 yr), 36 patients (58%) had evidence of metastases at diagnosis (stage range: T3N0M0-T4N1M1). Fifty patients out of 61 (82%) had a Gleason score from initial prostate histology. Among patients with a reported Gleason score, 31/50 patients (62%) had disease with a Gleason score ≥8. The median follow up was 40 mo (range, 31–52 mo; Table 1).

**Table 1:** Cohort demographics and clinical parameters.

*N* = 61	Age (yr)	Weight (kg)	Prostate volume (cm^3^)	PPF volume (cm^3^)	Initial PSA (ng/ml)	Nadir PSA (ng/ml)	Gleason score	Follow-up post-ADT (mo)
Median	75	77	36.71	24.80	40.30	1.1	4 + 4	40
Min.	53	44	13.32	7.45	1.30	0.1	3 + 3	31
Max.	89	111	164.50	139.41	653.9	61.3	5 + 5	52

ADT = androgen deprivation therapy; Max. = maximum; Min. = minimum; PPF = peri-prostatic fat; PSA = prostate-specific antigen.

### 3.1 PPFV is higher in patients who subsequently developed CRPC

**Fig. 1:**
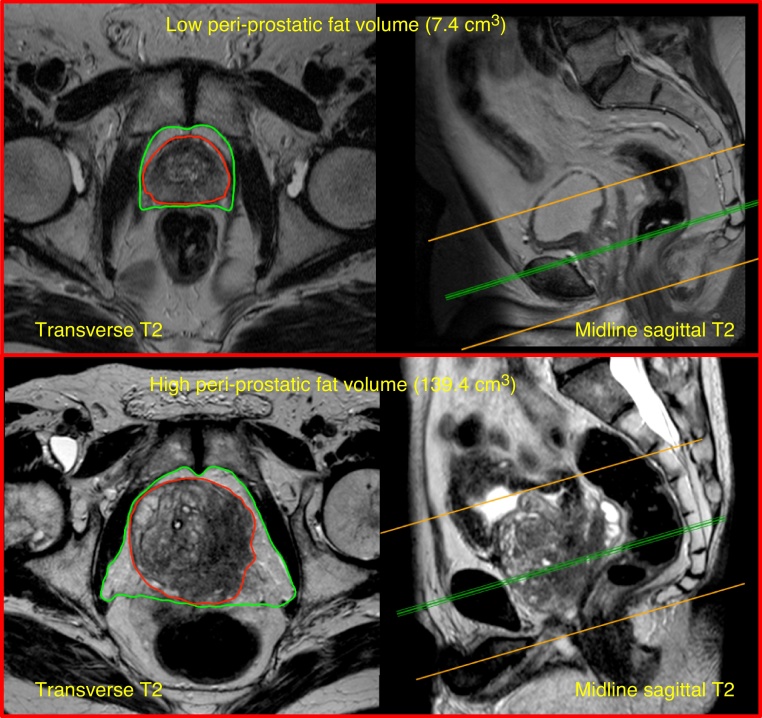
Pelvic magnetic resonance imaging scans of the lowest and highest peri-prostatic fat volume (PPFV) cases. Examples of T2-weighted pelvic magnetic resonance imaging scans of the lowest PPFV (7.4 cm^3^) and highest PPFV (139.4 cm^3^) cases within our cohort. Transverse (with regions of interest marked) and midline sagittal views (with cross-section reference line) are shown with the prostate highlighted in red and the surrounding peri-prostatic fat in green.

**Fig. 2:**
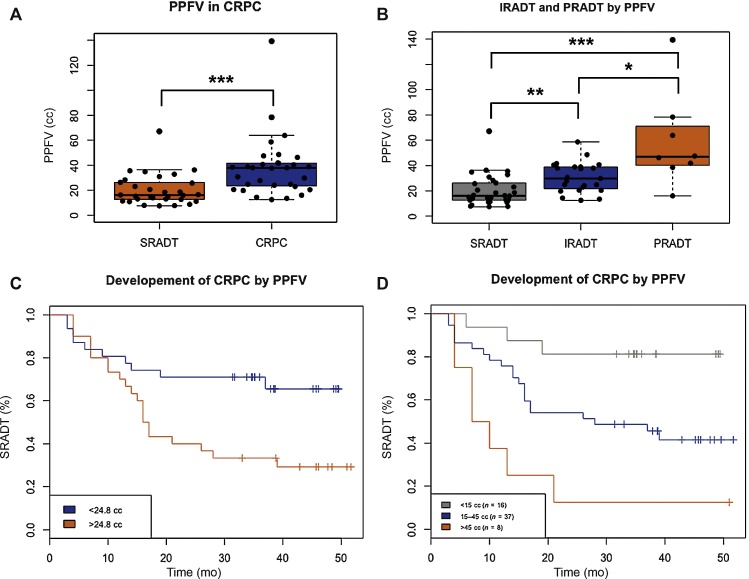
(A) Peri-prostatic fat volume (PPFV) in patients who develop castration resistant prostate cancer (CRPC). PPFV in patients who developed CRPC compared with patients with sustained response to androgen deprivation therapy (SRADT) with no evidence of rising prostate-specific antigen (PSA) within the follow-up period (median 40 mo), showing significantly greater PPFV in patients who develop CRPC within the follow-up period. All Box and Whisker diagrams middle bands represent the median value, the upper and lower box represents the upper and lower quartiles, whiskers extend from the upper and lower quartiles by 1.5 × the interquartile range, individual patients are also plotted as solid black points overlaid on the boxplots and laterally separated by a wrapping corral to avoid over-lay of same Y axis values. (B) PPFV in patients with initial response and poor response to ADT (IRADT and PRADT). PPFV in patients who initially respond (IR) to ADT with a PSA drop below the normal range (4 ng/ml) but then develop CRPC (IRADT) have greater PPFV than patients with a sustained response (SR) to ADT (SRADT) who did not develop CRPC within the follow-up period (p < 0.001 Wilcoxon Rank Sum test). Patients who have PRADT by nadir PSA not falling below the normal range (4 ng/ml) appeared to have an even higher PPFV (p = 0.006, Wilcoxon Rank Sum test). Individual patients are plotted as solid black points overlaid on the boxplots. (C and D) Analysis of time to CRPC and its relationship to PPFV. Kaplan Meier analysis of time to CRPC for two groups of patients divided by the median PPFV (1C; n = 31 red >24.8 cm^3^, n = 30 blue <24.8 cm^3^). Patients with higher PPFV showed greater and faster development of CRPC as shown by Kaplan-Meier curves over a range of PPFV (1D). *** p < 0.001.

PPFV measurements ranged from 7.4 cm^3^ to 139.4 cm^3^ (Fig. 1 depicts examples of the highest and lowest PPFV cases; median 24.8 cm^3^) and was significantly higher in patients who developed CRPC within the study period (median of 37.9 cm^3^) than those with SRADT (median of 16.1 cm^3^, p < 0.0001, Wilcoxon Rank Sum test; Fig. 2A) and unaffected by normalisation to prostate volume. PPFV weakly correlated with initial PSA levels (r_s_ = 0.26, p = 0.04, Spearman’s Rank correlation), but not with time to nPSA (r_s_ = 0.04, p = 0.78), Gleason score (r_s_ = −0.03, p = 0.82) or tumour stage (r_s_ = −0.08, p = 0.52). PPFV did correlate with nPSA (r_s_ = 0.45, p = 0.0003) and on further stratification of patients with evidence of CRPC, patients showing IRADT had lower PPFV than those with PRADT (p = 0.006, Wilcoxon Rank Sum test; Fig. 2B). PPFV also negatively correlated with time to CRPC (r_s_ = 0.34, p = 0.008, Spearman’s Rank correlation). As expected, nPSA levels were significantly higher in the CRPC group than in non-CRPC patients (median, 2.10 vs 0.40 respectively, p = 0.0003, Wilcoxon Rank Sum test).

### 3.2 PPFV did not correlate to whole body measures of adiposity

We found no correlation between PPFV and surrogate whole body measures of adiposity such as body weight and waist circumference (weight range: 44–111 kg, median 77 kg; waist circumference range: 72–123 cm, median 93 cm; r_s_ = 0.13, p = 0.3, and r_s_ = 0.17, p = 0.18, Spearman’s Rank correlation). In this study, patient body weight did not correlate with time to CRPC (r_s_ = 0.02, p = 0.9, Spearman’s Rank correlation), or response to ADT (PRADT; p = 0.3, Wilcoxon Rank Sum test; Supplementary Fig. 1)

### 3.3 PPFV significantly predicted the risk of developing CRPC

The impact of PPFV on time to CRPC is illustrated by a Kaplan-Meier survival analysis (Fig. 2C), with the time to CRPC significantly different between patients with above and below the median PPFV (24.8 cm^3^). Furthermore, a dose effect of the PPFV was observed with higher PPFV progressively showing reducing time to CRPC when patients were grouped according to their PPFV (0–15 cm^3^, 15–45 cm^3^, >45 cm^3^; Fig. 2D).

Univariate Cox proportional hazards models of time to development of CRPC were performed using factors of PPFV, nPSA, evidence of (lymph node or distant) metastasis at diagnosis, waist circumference, body weight, Gleason score, initial PSA, BMI, and local T stage (Table 2). Factors were initially considered in isolation, and PPFV, metastasis status at diagnosis, and nPSA individually predicted CRPC. However, in our cohort, whole body measures such as weight, waist circumference, and BMI (when available) did not individually predict CRPC (Table 2). Factors were then added and removed sequentially to generate an optimised multivariate model consisting of PPFV and presence of metastasis at diagnosis (Wald test = 24.7 on 2 degrees of freedom, p = 4.325e–06; Table 3). A multivariate model was then performed incorporating all previously identified significant predictors of CRPC on univariate analysis (PPFV, metastasis [lymph node and/or distant] at diagnosis, and nPSA), showing that only PPFV and distant metastasis at diagnosis were independent predictors of time to CRPC (Table 4). Including tumour stage, Gleason score, waist circumference, and body weight in the multivariate model did not improve the model fit (Wald test = 23.6 on 8 degrees of freedom, p = 0.002674; Table 5).

**Table 2:** Univariate analysis of known predictors of time to CRPC. Univariate Cox proportional hazards models of factors which may predict castrate resistant prostate cancer (CRPC) in our cohort. Showing hazard ratios, 95% lower and upper confidence intervals, *p* value, and number of observations. Factors have been ordered by their *p* value lowest to highest for their individual prediction of CRPC. Peri-prostatic fat volume (PPFV), nadir prostate-specific antigen (PSA), metastasis at diagnosis, and nodal disease at diagnosis are all individually significant predictors of CRPC. PPFV is grouped into 10 cm^3^ levels, metastasis at diagnosis is a binary factor (y/n), local T stage is divided into levels (T1c, T2, T3, T3a, T3b and T4), Gleason score is a binary factor greater than 4 + 4 (y/n). PSA values, body weight, waist circumference, and body mass index (BMI) are continuous variables.

	Hazard ratio	Lower confidence interval	Upper confidence interval	*p* value	*n*
PPFV (10 cm^3^)	1.408	1.211	1.637	<0.0001***	61
Nadir PSA (ng/ml)	1.058	1.023	1.095	0.00108**	59
Metastasis at diagnosis (y/n)	3.329	1.43	7.754	0.00529**	61
Lymph nodes at diagnosis (y/n)	2.617	1.168	5.865	0.0195*	59
Waist circumference (cm)	1.023	0.9887	1.059	0.189	61
Body weight (kg)	1.011	0.9842	1.039	0.418	60
Gleason score (>4 + 4)	0.8207	0.3765	1.789	0.619	50
BMI (Kg/m^2^)	1.039	0.8774	1.23	0.657	24
Local T stage (T1c–T4)	0.9638	0.7305	1.272	0.794	60
Initial PSA (ng/ml)	0.9998	0.9969	1.003	0.894	61

n = no; y = yes.

* *p* < 0.05.

** .

*** *p* < 0.0001.

**Table 3:** Optimised multivariate analysis of predictors of time to castrate resistant prostate cancer. Optimised multivariate Cox proportional hazards model included only peri-prostatic fat volume (PPFV) and metastasis at diagnosis. Showing hazard ratios, 95% lower and upper confidence intervals, *p*-value, and number of observations for PPFV and distant metastasis at diagnosis. Addition of further variables did not improve the model (Wald test = 24.7 on 2 degrees of freedom, *p* = 4.325e–06).

	Hazard ratio	Lower confidence interval	Upper confidence interval	*p* value	*n*
PPFV (10 cm^3^)	1.380	1.183	1.609	<0.00001**	61
Metastasis at diagnosis (y/n)	3.059	1.299	7.206	0.0105*	61

n = no; y = yes.

* *p* <0.05.

** *p* <0.0001.

**Table 4:** Multivariate analysis of significant predictors of time to castrate resistant prostate cancer. Multivariate Cox proportional hazards model of peri-prostatic fat volume (PPFV), distant metastasis at diagnosis, lymph nodes at diagnosis, and nadir prostate-specific antigen (PSA). The optimised multivariate model of PPFV and metastasis at diagnosis was not improved by the additional factors of lymph nodes at diagnosis or nadir PSA. PPFV prediction of castrate resistant prostate cancer remains independent after controlling for all other significantly predicting factors on univariate analysis (distant metastasis or lymph nodes at diagnosis and nadir PSA; Wald test = 27.5 on 4 degrees of freedom, *p* = 1.575e–05).

	Hazard ratio	Lower confidence interval	Upper confidence interval	*p* value	*n*
PPFV (10 cm^3^)	1.332	1.1198	1.584	0.0012**	57
Metastasis at diagnosis (y/n)	2.495	0.8526	5.080	0.0461*	57
Lymph nodes at diagnosis (y/n)	2.081	1.168	5.865	0.1074	57
Nadir PSA (ng/ml)	1.021	0.9774	1.066	0.3570	57

n = no; y = yes.

* *p* < 0.05.

** *p* < 0.0001.

**Table 5:** Multivariate analysis including expected predictors of time to castrate resistant prostate cancer (CRPC). Multivariate Cox proportional hazards model of peri-prostatic fat volume (PPFV), distant metastasis at diagnosis, lymph nodes at diagnosis, nadir prostate-specific antigen (PSA), Local T stage, Gleason score, waist circumference, and body weight. This multivariate model utilised previously identified factors reported to predict CRPC. PPFV prediction of CRPC remained independent after controlling for other factors previously predicting CRPC, however the model fit is not improved by them. (Wald test = 23.6 on 8 degrees of freedom, *p* = 0.002674).

	Hazard ratio	Lower confidence interval	Upper confidence interval	*p* value	*n*
PPFV (10 cm^3^)	1.2773	1.0474	1.558	0.0157*	47
Metastasis at diagnosis (y/n)	1.5728	0.5472	4.520	0.4005	47
Nodes at diagnosis (y/n)	3.4844	1.1519	10.540	0.0271*	47
Nadir PSA (ng/ml)	1.0246	0.9710	1.081	0.3752	47
Local T stage (T1c–T4)	0.8560	0.5762	1.272	0.4416	47
Gleason score (>4 + 4)	0.8706	0.2970	2.552	0.8006	47
Waist circumference (cm)	1.0642	0.9861	1.148	0.1097	47
Body weight (kg)	0.9590	0.9053	1.016	0.1547	47

n = no; y = yes.

* *p* < 0.05.

### 3.4 PPFV improved on current predictive factors known prior to ADT

**Fig. 3:**
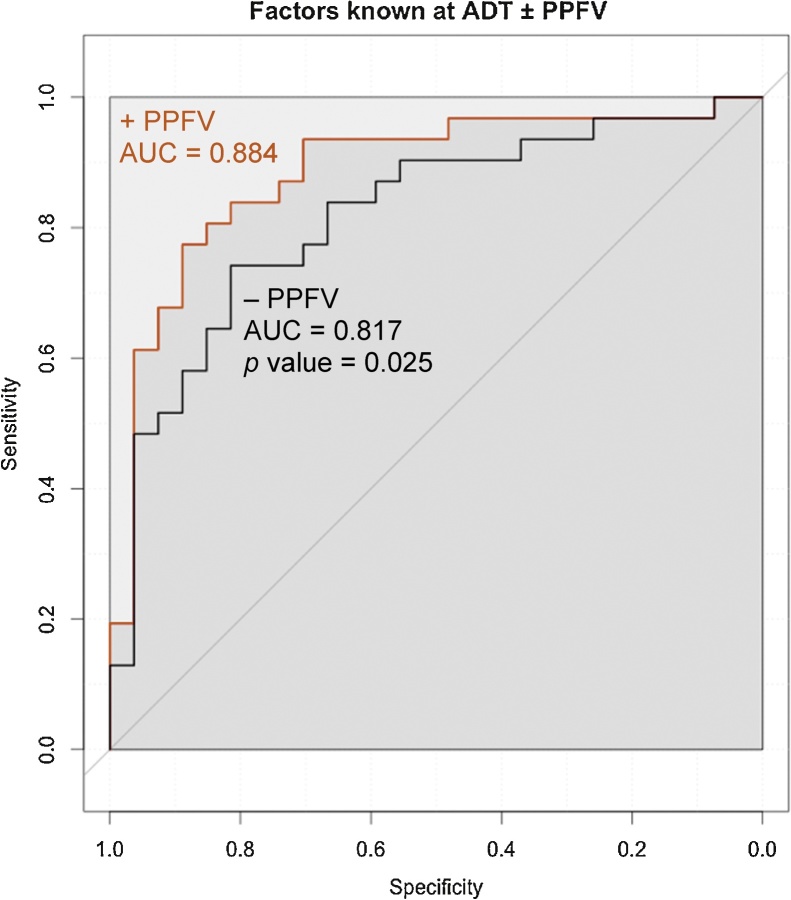
Receiver operating characteristics analysis curves of peri-prostatic fat volume (PPFV) added to current staging parameters known prior to androgen deprivation therapy (ADT). Receiver operating characteristics analysis of the multivariate model presented in Table 6 consisting of factors known at ADT (not including nadir prostate-specific antigen). Upper curve (red) shows prediction of the multivariate model in our cohort including PPFV with area under the curve (AUC) = 88.4%. Removing PPFV from the multivariate model in Table 6 produces the lower curve and AUC is significantly reduced to 81.7% (p = 0.02518, Delong’s test), suggesting a benefit of including PPFV in the multivariate model of factors known prior to commencing ADT in order to better predict the risk of developing castration resistant prostate cancer.

A multivariate Cox proportional hazards model was generated using the following factors that are known prior to ADT: PPFV, (nodal and distant) metastasis status at diagnosis, local T stage, initial PSA, and body weight (Table 6). The inclusion of PPFV into the panel significantly improved the performance of the prognostic model, with significantly better receiver operating characteristics area under curve (AUC; 88.4% and 81.7%, respectively, Delong’s test p = 0.025; Fig. 3). While Gleason score was not included in the model due to the limitation of the missing values of Gleason score in our cohort (11 out of 61 patients did not have an initial Gleason score), the addition of Gleason score in the model had little effect on the AUC (Supplementary Fig. 3).

**Table 6:** Multivariate model of factors known at the time of androgen deprivation therapy. Multivariate Cox proportional hazards model was generated using only factors known at androgen deprivation therapy. The highest area under the curve (AUC) was calculated for the predictive model including peri-prostatic fat volume (PPFV), metastasis at diagnosis, lymph nodes at diagnosis, local T stage, initial prostate-specific antigen (PSA), and body weight. Receiver operating characteristic analysis of the predicted model produced AUC 88.4% (confidence interval: 79.2–97.6%). PPFV removal from the multivariate model prediction significantly reduced the AUC to 81.7% (confidence interval: 70.56–92.88%, DeLong’s test *p*-value = 0.02518; Fig. 3).

	Hazard ratio	Lower confidence interval	Upper confidence interval	*p* value	*n*
PPFV (10 cm^3^)	1.3949	1.1826	1.6452	<0.0001***	58
Metastasis at diagnosis (y/n)	2.5702	1.5179	9.3923	0.00427**	58
Nodes at diagnosis (y/n)	3.3812	1.2910	7.4362	0.01135^*^	58
Local T stage (T1c–T4)	0.7093	0.4707	0.9100	0.01171*	58
Initial PSA (ng/ml)	0.9958	0.9922	0.9993	0.01798*	58
Body weight (kg)	1.0143	0.9757	1.0390	0.67004	58

n = no; y = yes.

* *p < *0.05.

** *p.*

*** *p < *0.0001.

The first 13 consecutive MRI scans were also analysed by a second observer to assess correlation and interobserver agreement (Bland Altman) of PPFV measures (Supplementary Fig. 2). High correlation was observed between independent measures: Two way intraclass correlation coefficient = 0.918, (p < 0.001, 95% confidence interval: 0.755–0.974). The median number of slices required for PPFV measurement was 6 (range, 4–11), time required to calculate PPFV as estimated to be 12 min (1 min × 2 regions × 6 slices) allowing 1 minute per region of interest with two regions of interest required for each slice (prostate and peri-prostatic fat region).

### 3.5 CCR3 expression was upregulated in tumours associated with high PPFV and CRPC

**Fig. 4:**
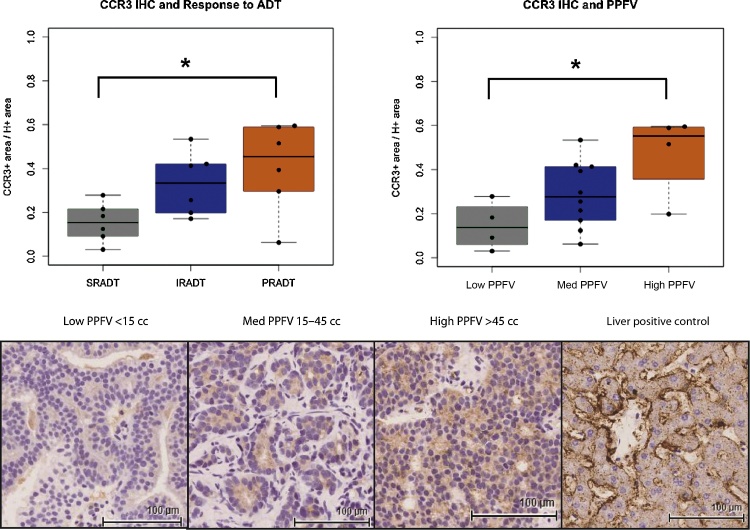
Analysis of chemokine receptor 3 (CCR3) immunohistochemistry (IHC) in relation to peri-prostatic fat volume (PPFV) and castration resistant prostate cancer (CRPC). (A) CCR3 stained area normalised by haematoxylin stained area (mm^2^; 3,3′-diaminobenzidine+/H+) was greater for patients who developed CRPC than patients who did not develop CRPC within the follow-up period (sustained response to androgen deprivation therapy [SRADT] vs initial response to ADT [IRADT] and partial response to ADT [PRADT] p = 0.04, Wilcoxon Rank Sum test). (B) CCR3 staining tended to be higher in patients with high PPFV (≥45 cm^3^, n = 4) compared with patients with a low PPFV (≤15 cm^3^, n = 4) with medium PPFV (15–45 cm^3^, n = 10) in-between suggesting a dose effect (low PPFV vs high PPFV, p = 0.057, Wilcoxon Rank Sum test). (C) Representative IHC stained images of low, medium (med) and high PPFV are also provided.

Immunohistochemistry for CCR3 was performed on prostate biopsy materials on randomly selected patient subcohorts according to their response to ADT: SRADT (n = 6), IRADT (n = 6), and PRADT (n = 6). CCR3 immunoreactivity was significantly upregulated in tumours from PRADT patients (p = 0.04, Wilcoxon Rank Sum test; Fig. 4A). There was a trend for progressive increase in CCR3 expression from low, medium, to high PPFV: low PPFV (<15 cc, n = 4), medium PPFV (15–45 cc, n = 10), and high PPFV (<45 cc, n = 4; low PPFV vs high PPFV, p = 0.057, Wilcoxon Rank Sum test; Fig. 4B). CCR3 immunohistochemistry in low, medium, and high PPFV patients are shown in Fig. 4C.

## 4 Discussion

Our report, for the first time, links clinical imaging to tumour-host interaction in the context of treatment resistance, supporting a role for PPF in CRPC. It is also in line with recent findings that local visceral fat functions through the CCR3/CCL7 axis in driving disease progression [10]. Hence, measurement of PPFV in patients undergoing primary ADT may help predict tumour response and the likelihood of developing CRPC. These measurements can be performed easily and reproducibly with basic training to recognise the relevant anatomy on MRI.

### 4.1 Study limitations and relationship of PPFV to current predictive factors of CRPC

In our cohort, initial PSA readings, tumour stage and high Gleason score, body weight, and waist circumference were not informative alone in predicting which patients will initially respond well to ADT. This may simply reflect the consequence of studying a relatively small patient cohort compared to n > 200 for studies showing an effect of these factors on the response to ADT [18]. The presence of nodal or distant metastasis at diagnosis and nPSA did predict CRPC, which is in keeping with current accepted practice that these are the best factors in predicting response to ADT. A limitation of our study is the relatively low number of patients (n = 61). However, inclusion criteria were limited to patients with MRI due to superior soft tissue contrast and delineation of the PPFV compared with computed tomography which is more commonly performed in advanced disease. Body weight and waist circumference are highly correlated (r_s_ = 0.76, p < 0.0001 Spearman’s rank correlation), therefore colinearity interactions are a limitation of the Cox proportional hazards model including both factors.

### 4.2 Future development of PPFV as a biomarker for CRPC

MRI measured PPFV significantly improves the AUC of the multivariate model of factors which can be known prior to ADT (excluding nPSA, as this can only be determined after ADT; Fig. 3). This suggests that the addition of PPFV measurement can be considered a useful biomarker of CRPC. The CHAARTED and STAMPEDE trials reported survival benefits of upfront chemo-hormonal therapy [5], but a key unmet clinical need remains in identifying patients who are likely to respond poorly to ADT and thus have the greatest benefit from upfront combined therapy prior to the development of CRPC.

The technique described provides an accessible and quick way to measure PPFV with acceptable interobserver agreement as well as good intraclass correlation. Future automated image analysis may reduce observer related variation in PPFV measurement. Our study suggests a relationship comparative to a dose response between the PPFV and tumour response to ADT. This is consistent with an important physiological role of the PPF in disease progression to CRPC. Besides the CCL7/CCR3 cytokine axis, other PPF-derived factors may also contribute to the local macro-environment to support PC progression including resistance to ADT [22].

## 5 Conclusions

Measurement of PPFV identifies patients at risk of biochemical relapse before commencing ADT. Current knowledge suggests that PPF contributes to the local tumour-promoting macro-environment, and PPFV may be considered a biomarker of such biological effects on tumours.

  ***Financial disclosures:*** Hing Y. Leung certifies that all conflicts of interest, including specific financial interests and relationships and affiliations relevant to the subject matter or materials discussed in the manuscript (eg, employment/affiliation, grants or funding, consultancies, honoraria, stock ownership or options, expert testimony, royalties, or patents filed, received, or pending), are the following: None.  ***Author contributions:*** Hing Y. Leung had full access to all the data in the study and takes responsibility for the integrity of the data and the accuracy of the data analysis.  *Study concept and design*: Salji, Hendry, Patel, Ahmad, Nixon, Leung.

*Acquisition of data*: Salji, Hendry, Patel, Ahmad, Nixon, Leung.

*Analysis and interpretation of data*: Salji, Hendry, Patel, Ahmad, Nixon, Leung.

*Drafting of the manuscript*: Salji, Hendry, Patel, Ahmad, Nixon, Leung.

*Critical revision of the manuscript for important intellectual content*: Salji, Hendry, Patel, Ahmad, Nixon, Leung.

*Statistical analysis*: Salji, Hendry, Patel, Ahmad, Nixon, Leung.

*Obtaining funding*: Salji, Hendry, Patel, Ahmad, Nixon, Leung.

*Administrative, technical, or material support*: Salji, Hendry, Patel, Ahmad, Nixon, Leung.

*Supervision*: Salji, Hendry, Patel, Ahmad, Nixon, Leung.

*Other*: None.  ***Funding/Support and role of the sponsor:*** None.  ***Acknowledgments:*** We would like to thank the multi-disciplinary team Cancer Audit Department, the Department of Radiology for the National Health Service Greater Glasgow and Clyde for access to magnetic resonance imaging scan data and all the patients involved in the study. Salji is a Medical Research Council Clinical Research Fellow (MR/L017997/1). Leung received research funding from Cancer Research UK (A15151).  
