# Supplementary figures and images for: Peri-prostatic Fat Volume Measurement as a Predictive Tool for Castration Resistance in Advanced Prostate Cancer
Source: Eur Urol Focus. 2018 Dec;4(6):858–66. doi: 10.1016/j.euf.2017.01.019 (PMC6314965; doi:10.1016/j.euf.2017.01.019)

**Supplementary Figure 1**


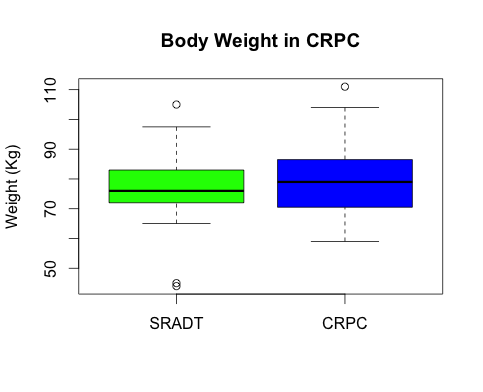

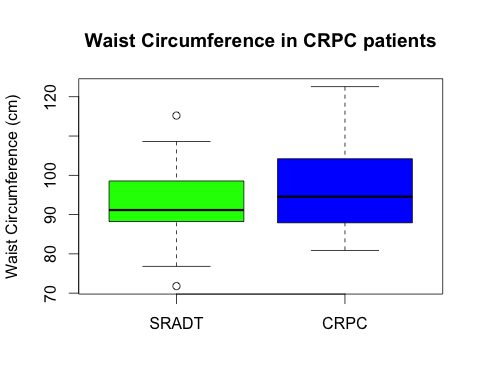

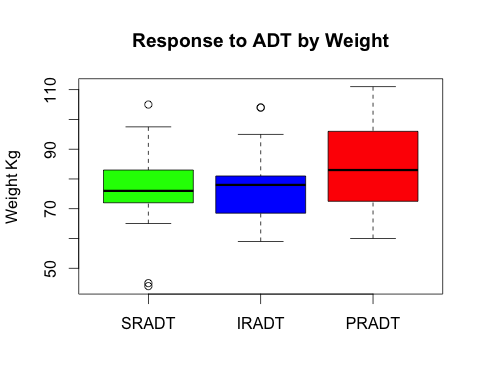

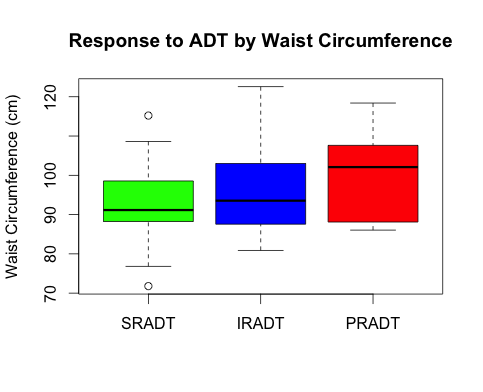

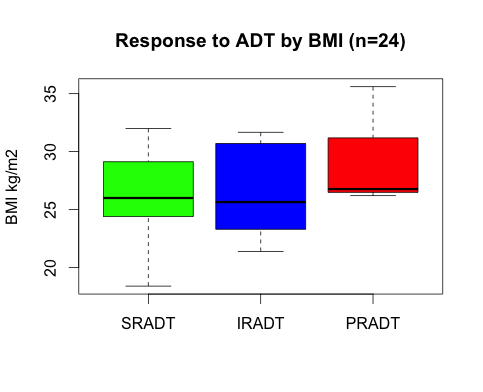

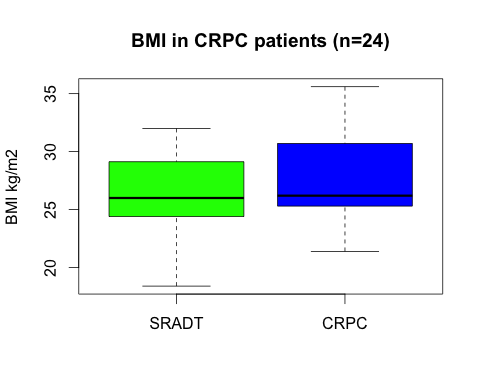


**Supplementary Figure 2**


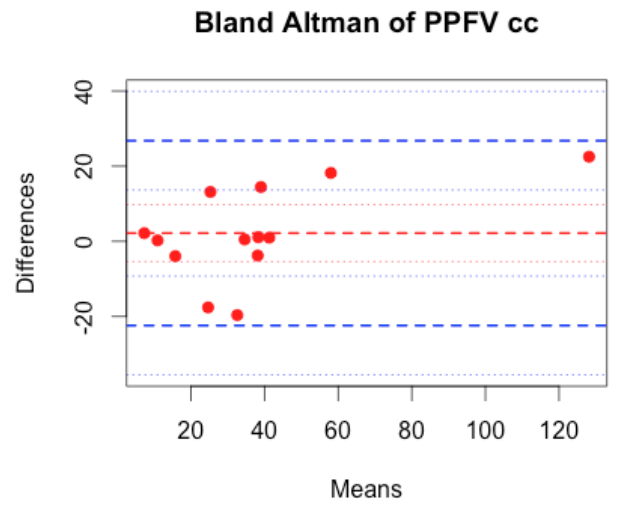


**Supplementary Figure 3**


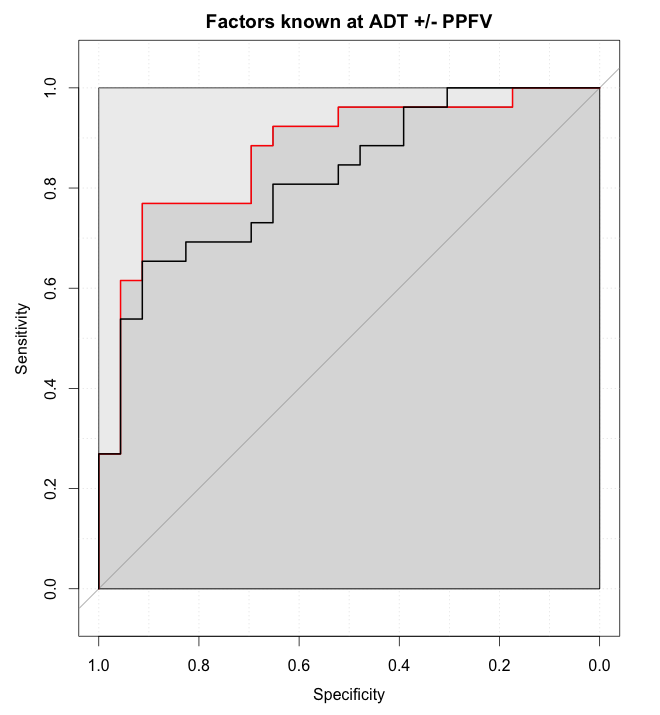


+ PPFV

AUC = 0.873

- PPFV

AUC = 0.821

p-value = 0.052

Supplement: Supplementary file 1 [file mmc1.docx]
