# Supplementary material for: Peri-prostatic Fat Volume Measurement as a Predictive Tool for Castration Resistance in Advanced Prostate Cancer
Source: Eur Urol Focus. 2018 Dec;4(6):858–66. doi: 10.1016/j.euf.2017.01.019 (PMC6314965; doi:10.1016/j.euf.2017.01.019)
Supplement: Supplementary file 2 [file mmc2.docx]

**Legends to supplementary figures**

**Supplementary figure 1. Body weight in patients with initial (IRADT) and poor response (PRADT) to ADT.**

Body weight was not significantly greater in patients who developed CRPC (left) (p=0.3, Wilcoxon Rank Sum test) but trended towards significance in patients with a poor response to ADT (right) (p=0.08).

**Supplementary figure 2.** **Bland Altman plot of two blinded measures of PPFV in 13 cases.**

Bland Altman plot of mean (x axis) vs. difference (y axis) of two separate measures of PPFV. Intra class correlation coefficient (ICC) showed a good correlation between measurements (ICC=0.918, p<0.001, 95% CI 0.755 - 0.974). However the Bland Altman plot suggests that variation increases as the measurements of PPFV increase.

**Supplementary figure 3. ROC analysis curves of PPFV added to current staging parameters known prior to ADT including Gleason score.**

Receiver operating characteristics (ROC) analysis of the multivariate model presented in table 4 consisting of factors known at ADT (not including nPSA) including Gleason score (n=47). Upper curve (red) shows prediction of the multivariate model in our cohort including PPFV with AUC = 87.3%. Removing PPFV from the multivariate model produces the lower curve and AUC is significantly reduced to 82.1% (p=0.052, Delong's test), suggesting a benefit of including PPFV in the multivariate model of factors known prior to commencing ADT.
